# Transforming Growth Factor Alpha (TGFα) Regulates Granulosa Cell Tumor (GCT) Cell Proliferation and Migration through Activation of Multiple Pathways

**DOI:** 10.1371/journal.pone.0048299

**Published:** 2012-11-14

**Authors:** Cheng Wang, Xiangmin Lv, Chao Jiang, Crystal M. Cordes, Lan Fu, Subodh M. Lele, John S. Davis

**Affiliations:** 1 Departments of Obstetrics and Gynecology, Olson Center for Women's Health, College of Medicine, University of Nebraska Medical Center, Omaha, Nebraska, United States of America; 2 Department of Pathology and Microbiology, College of Medicine, University of Nebraska Medical Center, Omaha, Nebraska, United States of America; 3 Department of Biochemistry and Molecular Biology, College of Medicine, University of Nebraska Medical Center, Omaha, Nebraska, United States of America; 4 VA Medical Center, Omaha, Nebraska, United States of America; 5 Key Laboratory of Protein Chemistry and Developmental Biology of Education Ministry of China, College of Life Sciences, Hunan Normal University, Changsha, Hunan, China; Florida International University, United States of America

## Abstract

Granulosa cell tumors (GCTs) are the most common ovarian estrogen producing tumors, leading to symptoms of excessive estrogen such as endometrial hyperplasia and endometrial adenocarcinoma. These tumors have malignant potential and often recur. The etiology of GCT is unknown. TGFα is a potent mitogen for many different cells. However, its function in GCT initiation, progression and metastasis has not been determined. The present study aims to determine whether TGFα plays a role in the growth of GCT cells. KGN cells, which are derived from an invasive GCT and have many features of normal granulosa cells, were used as the cellular model. Immunohistochemistry, Western blot and RT-PCR results showed that the ErbB family of receptors is expressed in human GCT tissues and GCT cell lines. RT-PCR results also indicated that TGFα and EGF are expressed in the human granulosa cells and the GCT cell lines, suggesting that TGFα might regulate GCT cell function in an autocrine/paracrine manner. TGFα stimulated KGN cell DNA synthesis, cell proliferation, cell viability, cell cycle progression, and cell migration. TGFα rapidly activated EGFR/PI3K/Akt and mTOR pathways, as indicated by rapid phosphorylation of Akt, TSC2, Rictor, mTOR, P70S6K and S6 proteins following TGFα treatment. TGFα also rapidly activated the EGFR/MEK/ERK pathway, and P38 MAPK pathways, as indicated by the rapid phosphorylation of EGFR, MEK, ERK1/2, P38, and CREB after TGFα treatment. Whereas TGFα triggered a transient activation of Akt, it induced a sustained activation of ERK1/2 in KGN cells. Long-term treatment of KGN cells with TGFα resulted in a significant increase in cyclin D2 and a decrease in p27/Kip1, two critical regulators of granulosa cell proliferation and granulosa cell tumorigenesis. In conclusion, TGFα, via multiple signaling pathways, regulates KGN cell proliferation and migration and may play an important role in the growth and metastasis of GCTs.

## Introduction

Granulosa cell tumors (GCTs) account for 5–8% of all ovarian cancers [1]. GCTs present many features typical of normal granulosa cells. They express the FSH receptor gene, secret inhibins and produce estrogen [2], [3]. One-third to one-half of patients with GCTs develop endometrial hyperplasia and 8–33% develop endometrial adenocarcinoma due to the excessive estrogen produced by GCTs. Occasionally, these tumors may produce androgens leading to virilization and reproduction dysfunction [1], [4]. Clinically, GCTs are often slow to develop and have a propensity for late recurrence [5]–[6]. However, these tumors have malignant potential and about 50% of cases are diagnosed with metastases [7]–[8]. There are reported cases of lung, liver, brain, bone, diaphragm, abdominal wall, pancreas and adrenal gland metastases from GCTs [9]–[17]. The mechanisms underlying GCT initiation, progression, recurrence and metastasis are unknown.

Accumulating evidence suggests that these processes may involve the disruption of regulatory pathways that function during normal ovarian development, folliculogenesis, and ovulation [18], [19]. Granulosa cells are highly regulated by gonadotropins, steroid hormones and growth factors. Abnormal activities in the pathways activated by any of these factors may induce transformation of follicular granulosa cells and may promote GCT tumor growth, recurrence or metastasis. Among these factors, the epidermal growth factor (EGF) family of ligands and ErbB family of receptor tyrosine kinases are possible prominent contributors for GCT initiation and progression. ErbB family proteins play critical roles in the regulation of normal ovarian follicle development and ovulation [20]–[21]. EGF is produced in ovarian follicles [20], [22] and activation of the EGF receptor (EGFR) stimulates DNA synthesis and proliferation of granulosa cells in ovarian follicles, and modulates ovarian steroidogenesis and granulosa cell differentiation [20], [23]–[25]. It seems, therefore, that aberrant expression of ErbB family receptors and/or disrupted signal transduction may result in gene amplification and genetic mutations in ovarian cells and contribute to the development of malignant transformation of these cells [26]–[27]. There is abundant evidence that EGFR activation drives cellular processes linked to ovarian epithelial tumor development, tumor cell survival and metastasis; and clinical trials are ongoing to target ErbB family receptors for epithelial ovarian cancer therapy [27], [28]. Despite the advances in epithelial ovarian cancer research, the function of the EGF family ligands and ErbB family of receptors in GCTs is largely unknown.

Among EGF family ligands, TGFα is one of the most important local growth factors regulating follicle development and tumorigenesis [29]. TGFα shares only about 30% structural homology with EGF but can bind to the EGF receptor with similar affinity and signals via EGFR [29]. TGFα is found in granulosa cells of preantral follicles and theca cells of healthy human preantral, antral and preovulatory follicles [30]–[32]. Atretic follicles and theca lutein cells are also strongly positive for TGFα [30]–[32]. TGFα, not EGF, is also present in human follicular fluid. The EGFR is observed in human granulosa cells of antral follicles [32]. In bovine and porcine ovarian follicles, TGFα is produced by mesenchymal/stromal-derived theca cells of the follicle and act primarily on the epithelial granulosa cells [33]–[34]. These observations support the notion that TGFα plays a more pronounced role than EGF in the regulation of granulosa cell function.

The mitogenic action of TGFα has been implicated in growth, progression and outcome of epithelial ovarian cancer. TGFα is associated with rapidly growing fetal tissues and neoplastic cells from a number of postnatal and adult tissues [35]–[39]. Antonio et al. [40] reported that 92% of primary ovarian tumors express immunoreactive TGFα. TGFα present in effusions from cancer patients is also associated with tumor burden in patients with epithelial ovarian cancer [41]–[42]. Additionally, TGFα has been reported to regulate secretion of CA125, a common serum marker of ovarian cancer, and tissue plasminogen activator from human ovarian carcinomas [43]. However, whether TGFα is involved in the regulation of GCT growth, progression and metastasis is still an open question.

Studies on GCT biology and therapy have been limited by the lack of an appropriate experimental cellular model. Due to the relatively rare occurrence of GCTs, few human granulosa tumor cell lines have been established [44], [45]. The KGN and COV434 GCT cell lines are two useful cell lines for the study of GCTs because they retain many features of primary GCT cells [44], [45], [46]. The KGN cell line was derived from a 73-yr-old patient with a recurrent, metastatic GCT in the pelvic region [3], [44]. The COV434 cell line was derived from a 27-yr-old patient with a metastatic GCT [46]. Evidence suggests that the COV434 and KGN cell lines are derived from juvenile and adult GCTs, respectively [46]. Both cell lines maintain many of the physiological features of ovarian granulosa cells. They have detectable aromatase activity and can produce estrogen. They express FSH receptors and can respond to FSH stimulation [3]. Their steroidogenic activities are also similar to that of the granulosa cells. A recent study reported that advanced passage KGN cells grow faster and are more invasive than early passage cells [44]. Therefore, KGN cells appear to be a useful cell line for the study of the growth and metastasis of GCTs. In the present study, we determined the expression of ErbB family receptors in human granulosa cell tumor tissues and the COV434 and KGN cell lines. We used the KGN cell as a cellular model to determine the action of TGFα on cell cycle progression, cell proliferation and migration. We also determined the signaling pathways that mediate TGFα actions in KGN cells.

## Materials and Methods

### Chemicals, cells and human granulosa cell tumor tissue slides

TGFα and EGF were from R&D systems Inc. (Minneapolis, MN). DMEM and other cell culture medium were from Invitrogen (Carlsbad, CA). Fetal bovine serum (FBS) was from HyClone laboratories Inc. (Logan, UT). Ribogreen RNA quantification kit and Alexa-conjugated secondary antibodies were from Molecular Probes, Inc. (Eugene, OR); wortmannin, U0126, rapamycin and other kinase inhibitors were from Calbiochem (San Diego, CA). The RNeasy Mini Kit was from QIAGEN Inc. (Valencia, CA). Antibodies against EGFR (ErbB1) and phosphorylated forms of EGFR, ERK-1/2, Akt, P38, p70S6K, S6, mTOR and rictor were from Cell Signaling Technology Inc. (Danvers, MA). Antibodies against ErbB2 were from Abcam (Cambridge, MA). Antibodies against β-tubulin, β-actin and BrdU were from Sigma (St. Louis, MO). Antibodies against ErbB3, ErbB4 and non-phosphorylated Akt and ERK1/2 were from Santa Cruz (Santa Cruz, CA). Secondary antibodies for Western blotting chemiluminescence were from Jackson Immunoresearch Laboratories Inc. (West Grove, PA); enhanced chemiluminescence (ECL) Advance™ Western Blotting Detection kit was from Amersham Bioscience (Piscataway, NJ); Optitran nitrocellulose transfer membrane was from Schleicher&Schuell Bioscience (Dassel, Germany); PCR chemicals were from Roche Molecular Biochemicals (Indianapolis, IN), Amersham Pharmacia Biotech Boehringer (Piscataway, NJ), and Promega Corp. (Madison, WI). All other molecular-grade chemicals were purchased from Sigma (St. Louis, MO), Fisher (Pittsburgh, PA), or United States Biochemical (Cleveland, OH).

The human granulosa cell tumor cell line KGN was from the Riken Biosource Center (Ricken Cell Bank, Japan). The human COV434 and COV644 cell lines were generous gifts from Dr. CE van der Minne (University Hospital, Leiden, The Netherlands). The SKOV-3 cell line was from ATCC (Manassas, VA). Human ovarian granulosa cells were isolated from two medium size follicles (5–10 mm in diameter) of a 33 year-old patient who received oophroectomy for causes other than an ovarian disorder. Informed consent was obtained to use the ovarian tissue for research. The cells were isolated manually with a needle and cultured in DMEM supplemented with 5% FBS. Granulosa cells were passaged once prior to isolating protein or RNA. Preexisting human granulosa cell tumor slides were obtained from the Department of Pathology, University of Nebraska Medical Center (UNMC). All research involving human tissue was approved by the University of Nebraska Medical Center Institutional Review Board. Data were analyzed anonymously.

### Immunohistochemical detection of ErbB family receptors in granulosa cell tumors

Formalin-fixed, paraffin-embedded tissue slides were stained with standard streptavidin-biotin immunoperoxidase methods with EGFR, ErbB2, ErbB3 and ErbB4 antibodies. Briefly, tissue sections were deparaffinized in xylene and rehydrated using a standard alcohol series. Sections were then immersed in 0.1 M sodium citrate solution and heated to 100°C for antigen retrival. Endogenous peroxidase activity was blocked by incubation in hydrogen peroxide (0.3%) in phosphate buffered saline (PBS) for 10 minutes at room temperature (RT). The sections were blocked with 10% normal donkey serum at RT for 1 hour. Primary antibodies were applied overnight at 4°C. After washing away unbound primary antibodies with PBS, biotinylated secondary antibody and streptavidin peroxidase complex (DAKO LSAB Kit, Carpinteria, CA) were added consecutively for 10 minutes at RT and washed in PBS. Peroxidase activity was visualized with a DAB kit (Invitrogen, Carlsbad, CA). The sections were then washed and counterstained with Mayer's hematoxylin. For controls, primary antibodies were replaced by blocking buffer containing the same amount of non-immune IgG from the host species. The most representative areas fulfilling the established histologic criteria were selected and evaluated under a light microscope (Leica, Germany).

### Protein expression of ErbB family receptors in ovarian cancer cell lines

To determine the expression of the ErbB family of receptors, cultured cells were directly lysed in the dishes, homogenized by sonicating in 100 µl of EGFR-buffer (10 mM Tris pH 7.4, 100 mM NaCl, 1 mM EDTA, 1 mM EGTA, 1 mM NaF, 20 mM Na_4_P_2_O_7_, 1% triton X-100, 10% glycerol, 0.1% SDS and 0.5% deoxycholate) with protease inhibitor cocktails and PMSF and kept on ice for 20 minutes. The homogenate was centrifuged at 14,000 g at 4°C for 15 minutes. The supernatant was collected and the protein was measured with Micro BCA™ Protein Assay Kit (PIERCE, Rockford, IL) according to the manufacturer's instructions. Protein (20 µg) was fractioned with 10% polyacrylamide gels, electrotransferred to Optitran membrane and probed overnight at 4°C with primary antibodies against EGFR, ErbB2, ErbB3 and ErbB4. Peroxidase conjugated donkey anti-rabbit or peroxidase conjugated donkey anti-mouse secondary antibodies (Jackson ImmunoResearch Laboratories, Inc., West Grove, PA) were applied to the membrane and the bound secondary antibody was detected with the GE Healthcare Amersham ECL Plus Western Blotting Detection Reagents (Piscataway, NJ). The signal was recorded by a UVP gel documentation system (UVP, Upland, CA). β-tubulin was used as a loading control. Each group had at least three replicates of samples collected from three different experiments.

### Localization of ErbB family receptors by fluorescence immunohistochemistry

To localize the ErbB family of receptors, KGN cells were seeded on coverslips and cultured to 80% confluence. Cells were fixed in freshly prepared ice-cold 4% paraformaldehyde in PBS (pH 7.4) for 10 minutes, washed with PBS, blocked with 10% normal donkey serum and probed overnight at 4°C with antibodies against EGFR, ErbB2, ErbB3 and ErbB4. Antigens were visualized by applying Alexa-488-conjugated donkey anti-rabbit or donkey anti-mouse secondary antibodies. The nucleus was stained with DAPI. Images were captured with a LSM 710 Confocal Laser Scanning Microscope equipped with an AxioCam MRc 5 and analyzed with a Zeiss Zen 2009 software (Carl Zeiss Microscopy, Thornwood, NY). The exposure time of the camera was set for subtracting background fluorescence that was present in sections incubated with the non-immune IgG of the host species. Specific fluorescence signals (immune-signals) were merged with their corresponding nuclear signals to determine the cellular site of protein expression.

### mRNA expression of the ErbB family of receptors, EGF and TGFα

The expression of mRNA for ErbB receptor family members in primary cultures of human granulosa cells, KGN and COV434 GCT cells was detected by RT-PCR. SKOV-3 (ErbB2 overexpressing cells) and COV644 cells (ErbB4 negative cells) were used as positive and negative controls. Primary cultures of normal human granulosa cells were used as a positive control for the detection of EGFR and TGFα [47]. Total RNA was isolated from cultured cells using the RNeasy Mini Kit (QIAgen Inc., Valencia, CA) according to the manufacturer's instructions. The amount of RNA was quantified using a Ribogreen RNA quantification kit (Molecular Probes) according to the manufacturer's protocol. The sequences of primers used in this study are presented in Table 1. PCR primers were synthesized in the Eppley DNA synthesis Core Facility (University of Nebraska Medical Center, Omaha, NE). Aliquots of reverse transcribed product from each cell line were used for RT-PCR. PCR reactions were continued for 30 cycles after an initial denaturation at 95°C for 15 minutes to activate the HotStart Taq polymerase (QIAGEN). Each cycle of PCR consisted of 30 seconds of denaturation at 94°C, 30 seconds of annealing at a specific temperature (see Table S1), 30 seconds of extension at 72°C and ending with a 10 minute final extension at 72°C at the completion of the 30th cycle. PCR products were loaded onto a agarose gel and separated by electrophoresis. β-actin was used as a loading control.

**Table 1:** Effect of TGFαtreatment on cell cycle progression^1^.

Cell Cycle Phase	Control	TGFα	*P*
S phase	10.9±0.3%	23.2±2.3%	*P*<0.001
G2/M phase	6.5±1.4%	13.8±0.8%	*P*<0.05
G1 phase	82.6±1.6%	63.2±3.2%	*P*<0.001

1 , KGN cells were treated in serum-free media without (Control) or with TGFα (10 ng/ml) for 24 hours. Cell cycle analysis was performed by flow cytometry. Data are expressed as a percentage of cells in each phase of the cell cycle. Shown are means ± SEM from 3 separate experiments.

### Cell cycle analysis

KGN cells were cultured with or without TGFα (10 ng/ml) for 24 hours. Cells were then trypsinized, fixed and permeabilized with 70% ethanol overnight at −20°C. Cells were then labeled with propidium iodide for 30 minutes at 37°C and flow cytometry was used to determine the cell cycle distribution of the KGN cells. Inhibitors of TGFα-stimulated cell signaling pathways [AG1478 (EGFR tyrosine kinase inhibitor, 100 nM), U0126 (MEK1/2 inhibitor, 4 µM), LY294002 (PI3 kinase inhibitor, 100 nM), wortmannin (PI3 kinase inhibitor, 100 nM), or rapamycin (mTOR inhibitor, 20 nM)] were used to determine which pathway(s) participate in the effect of TGFα on KGN cell cycle progression. KGN cells were cultured as described above with or without TGFα (10 ng/ml) and/or kinase inhibitors for 24 hours. The pathway inhibitors used in these experiments did not affect cell survival, as shown by the flow cytometry data.

### Cell proliferation and viability assays

To determine the effect of TGFα on KGN cell proliferation, 30% confluent KGN cells were incubated in DMEM with different concentrations of FBS (0%, 5% and 10%) in the presence or absence of TGFα (10 ng/ml) for 48 hours. Inhibitors of TGFα-stimulated cell signaling pathways, AG1478 (100 nM), U0126 (4 µM), LY294002 (100 nM), wortmannin (100 nM), or rapamycin (20 nM) were used to determine which pathway(s) participate in the response TGFα on KGN cell proliferation. On completion of the incubation, cultures were typsinized and cell numbers were determined with an Invitrogen Countess® Automated Cell Counter (Carlsbad, CA).

The MTT assay was used to determine the effect of TGFα on KGN cell viability. Cells were plated in 24-well plates and incubated in media containing 10% FBS to approximately 30% confluence. Cells were then cultured in serum-free media and treated with or without TGFα in the presence or absence of the above mentioned pathway kinase inhibitors for 48 hours. MTT assay was performed with a Vybrant® MTT Assay Kit (city, state) according to the manufacturer's instruction.

The BrdU incorporation method was also used to determine the effect of TGFα on DNA synthesis. KGN cells were cultured on coverslips in media containing 10% FBS to 30% confluence and then cultured in serum-free media before treating with or without TGFα (10 ng/ml) for 36 hours. BrdU (10 mM) was added to the culture medium 3 hours before culture termination. The cultured cells were harvested, fixed in 4% paraformadehyde at 4°C for 10 minutes, and then treated with 2 M HCl at 37°C for 20 minutes. After blocking with 10% normal donkey serum at RT for 1 hour, cells were incubated with anti-BrdU antibody (Sigma) at 4°C for 16 hours. After washing with PBS, the cells were probed with Alexa-488-conjugated donkey anti-mouse antibody at RT for 30 minutes. The images were captured by a Q-imaging digital camera and Openlab image analysis software and BrdU positive cells were quantified.

### Western blot detection of the effect of TGFα on the activation of signaling pathways in KGN cells

To determine the effect of TGFα on the activation of signaling pathways, KGN cells were cultured in media containing 10% FBS to ∼70% confluence and then cultured in serum-free media before treating with or without TGFα (10 ng/ml) for 10, 30, 60, or 120 minutes. Cells were directly lysed and prepared as described above. The protein was measured with Micro BCA™ Protein Assay Kit and 20 µg of protein was fractioned with 10% polyacrylamide gels, electrotransferred to Optitran membrane and probed with antibodies against phosphorylated ERK1/2, Akt, mTOR, p70S6K, S6, Rictor, P38 and CREB, followed by a peroxidase conjugated donkey anti-rabbit secondary antibody (Jackson ImmunoResearch Laboratories, Inc, West Grove, PA). Secondary antibody binding was detected with ECL and the signal was recorded by a UVP gel documentation system (UVP, Upland, CA). After completely stripping the ECL signal of the phospho-proteins, membranes were re-probed with ERK1/2, Akt1, mTOR, CREB antibodies that recognize nonphosphorylated forms of proteins to confirm that TGFα specifically altered ERK and Akt activity but not its expression levels. β-actin was used as a loading control. Each group had at least three replicates of samples collected from three cultures performed on separate days.

To screen pathways involved in the TGFα stimulation of KGN cell proliferation and migration, KGN cells were pre-treated with the pathway inhibitors indicated above for 3 hours prior to treatment with TGFα (10 ng/ml) for 10 minutes. Cells were then collected and analyzed with Western blotting as described above.

### Cell migration assay

The wound healing assay was used to determine whether TGFα regulated KGN cell motility. KGN cells were cultured in 6-well cell plates until confluent. Wounds were made by scratching the cellular layer with a 100 µl pipette tip. After washing away the cell debris, serum-free medium with or without TGFα (10 ng/ml) was added to the culture. Zero hour pictures (0 h-control) were taken for each “wound” with an Olympus inverted microscope equipped with a DP71 digital camera (Olympus America, Inc. Center Valley, PA). Cells were incubated for 20 hours and then another picture for each “wound” was taken. The “wound” area was measured with a computerized Microsuite™ FIVE imaging software (Olympus America, Inc. Center Valley, PA).

A chemotaxis assay was also used to confirm the effect of TGFα on KGN cell migration. KGN cells (4×10^5^) in 250 µl of serum-free DMEM with or without 10 ng/ml of TGFα were placed in a Transwell® insert (8 µm pore size, Corning-Costar, Lowell, MA). The inserts were then placed in wells of a 24-well plate containing 750 µl of DMEM-FBS (5%) and incubated at 37°C for 6 hours. After incubation, the cells on the top of the membrane were removed with a cotton swab. Cells which had migrated to the underside of the membrane were fixed and stained with 0.04% crystal violet in methanol for 30 minutes. Cells were then photographed (100× magnification) and 10 pictures per group were quantified with Openlab® software (http://www.improvision.com). Experiments were repeated three times and at least three inserts were used for each treatment group.

### Statistics

All cultures and immunofluorescent localization and Western blot experiments were repeated at least three times and representative images are presented. All quantitative data were analyzed using one-way ANOVA followed by Tukey's Multiple Comparison Test by applying GraphPad Prism Version 5.01 for Windows (San Diego, CA). Data are represented as means ± SEM. *P*<0.05 was considered significant.

## Results

### Expression of the ErbB family of receptors in human granulosa cell tumors and cell lines

**Figure 1:**
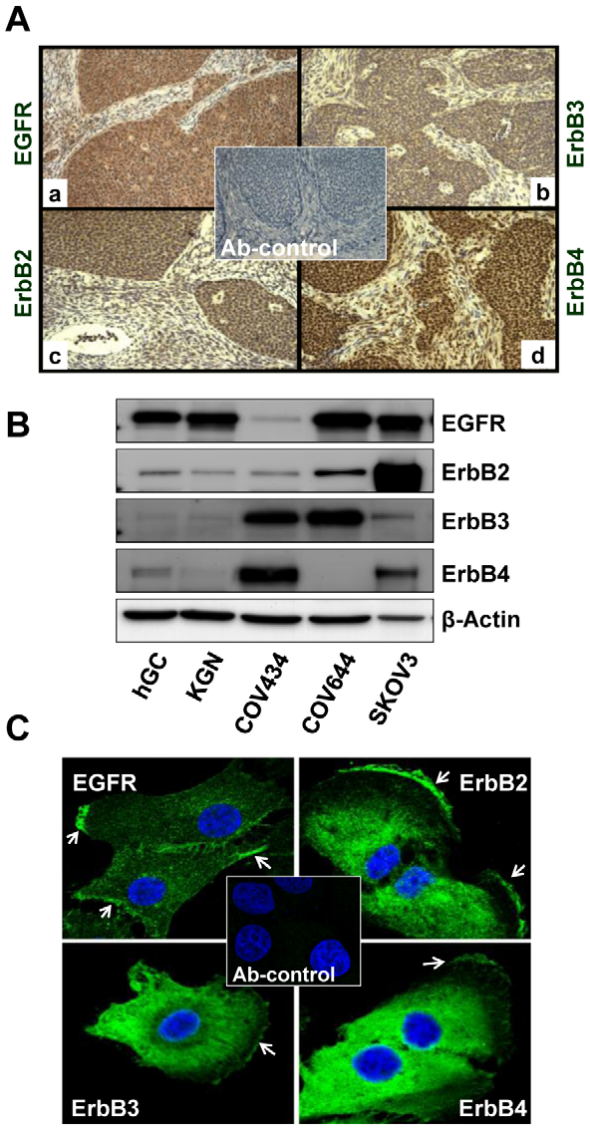
Expression of ErbB receptor family members in human ovarian granulosa cell tumors (GCTs). **A**) Immunohistochemistry was used to detect the expression of EGFR (a), ErbB2 (b), ErbB3 (c) and ErbB4 (d) in paraffin-embedded human GCT tissues. Magnification, 400×. **B**) Western blot detection of expression of ErbB family receptor proteins in human ovarian granulosa cells and GCT cell lines. SKOV-3 and COV644 cells were used as controls for the detection system. **C**) Fluorescent immunohistochemistry localization of ErbB receptors in KGN cells. The images were captured with a confocal laser scanning microscope. Arrows indicate the membrane localization of ErbBs in KGN cells. Magnification, 630×; Ab-control: 2^nd^ antibody only control, magnification, 200×.

Reports examining the expression of ErbB family members in ovarian GCTs are rare and the results are inconsistent [48]–[49]. In the present study, GCT tissue sections from 5 of 5 patients stained positive for EGFR, ErbB3 and ErbB4 (Figure 1A). No staining was detected in the control sections. The expression patterns of the four ErbB receptors were similar, with almost all positive signals localized to the GCT cells. Expression of ErbB receptors in the stroma was low or undetectable.

Western blot was used to detect the existence of ErbB family members in the GCT cell lines. Since human granulosa cells are known to express EGFR [47], we used primary cultures of human granulosa cells as a positive control. We also used SKOV-3 cells (ErbB2 overexpressing cells) and COV644 (ErbB4 negative) as positive and negative controls, respectively. Western blot analysis showed that human granulosa cells expressed EGFR, SKOV-3 cells expressed abundant ErbB2 expression, while COV644 cells were ErbB4 negative, validating our detection system (Figure 1B). Western blot results showed that the KGN cells and human granulosa cells had similar patterns of expression of the ErbB family members, with EGFR as the predominant receptor in these cells. However, in another proven GCT cell line, COV434 cells [46], the expression of ErbB3 and ErbB4 was dominant compared to EGFR and ErbB2 (Figure 1B).

Localization of ErbB family members with fluorescent immunohistochemistry and confocal laser scanning microscopy confirmed the expression of these receptors in the KGN cells. EGFR was localized mainly to the membrane of the cultured KGN cells, while ErbB2, ErbB3 and ErbB4 were mainly localized to the peri-nuclear area and the plasma membrane of KGN cells (Figure 1C).

**Figure 2:**
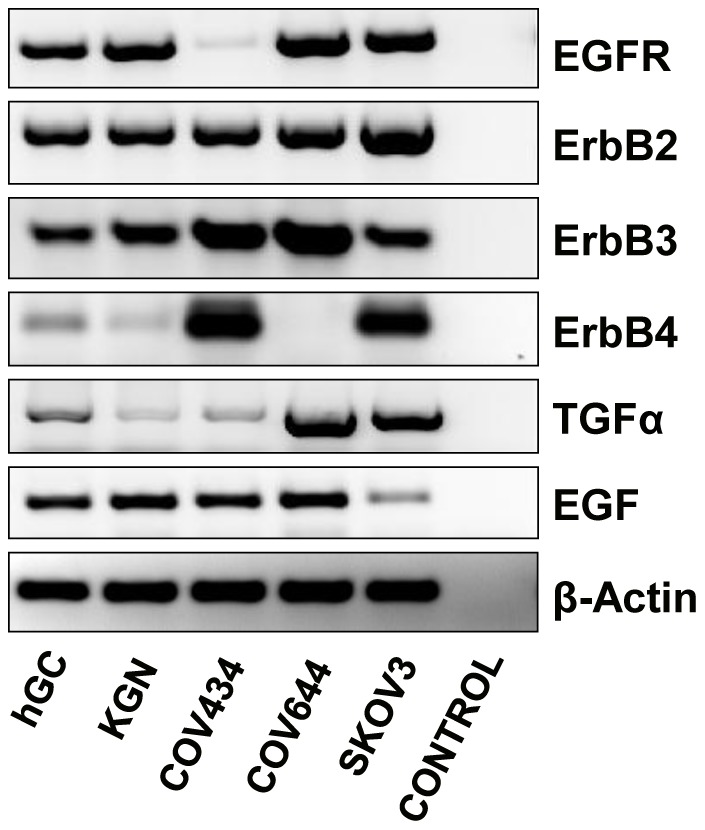
Expression of TGFα, EGF and ErbB family receptor mRNA in KGN and COV434 GCT cell lines. SKOV-3 (ErbB2 overexpressing cells) and COV644 cells (ErbB4 negative cells) were used as positive and negative controls. Primary cultures of normal human granulosa cells were used as a positive control for the detection of EGFR and TGFα. β-actin mRNA was used as an internal loading control.

Consistent with protein data, RT-PCR results showed that SKOV-3 cells have the highest ErbB2 expression while COV644 cells do not express ErbB4 mRNA. RT-PCR results showed that KGN and COV434 GCT cells express mRNA for EGFR, ErbB2, ErbB3 and ErbB4 (Figure 2). The KGN cells and primary cultures of human granulosa cells had comparable mRNA for each of the ErbB receptors. However, COV434 cells had high levels of ErbB3 and ErbB4 mRNA and lower levels of EGFR mRNA compared with that of KGN cells. The mRNA for EGFR ligands, EGF and TGFα, were also expressed in the KGN and COV434 cell lines (Figure 2).

### TGFα Stimulates GCT cell proliferation

**Figure 3:**
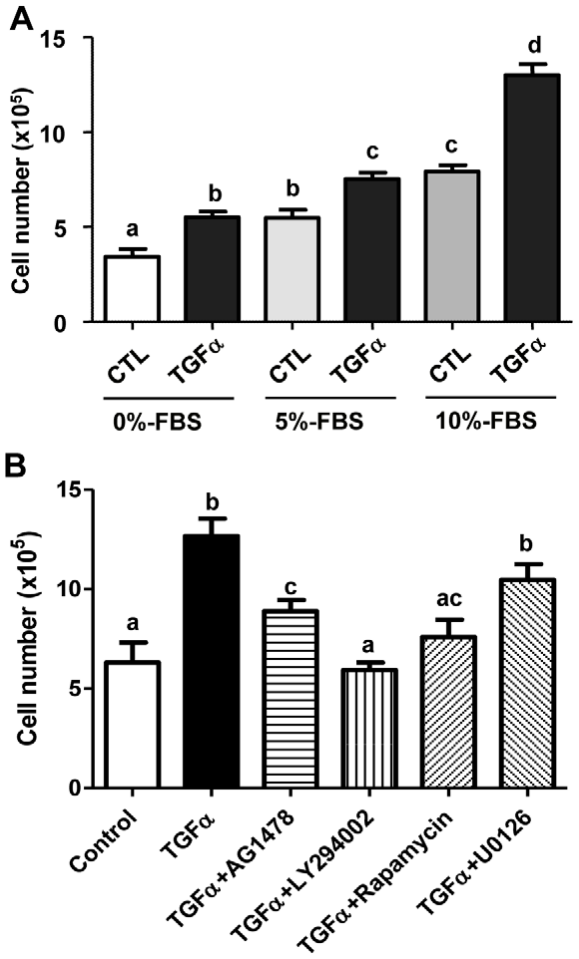
Effect of TGFα on KGN cell proliferation. **A**) KGN cells were treated without (CTL) or with TGFα (10 ng/ml) in DMEM supplied with increasing amounts of serum for 48 hours and cell numbers were counted. **B**) KGN cells were treated without (Control) or with TGFα and/or different kinase inhibitors in DMEM for 48 hours and cell numbers were counted. Bars represented means ± SEM, Bars with different letters are significantly different (*P*<0.05) from each other. CTL = control; AG1478 (100 nM): EGFR kinase inhibitor; U0126 (4 µM): MEK inhibitor; Rapamycin (20 nM): mTOR inhibitor; LY294002 (100 nM), PI3K inhibitor.

We used the well-characterized KGN GCT cell line [44], [46] to study the possible role of TGFα in GCT progression. KGN cells were cultured without (Control) or with TGFα (10 ng/ml) for 48 hours in DMEM with different concentrations of serum (Figure 3A). Treatment of KGN cells with TGFα in serum-free DMEM resulted in a statistically significant increase in KGN cell number compared to control (*P*<0.05). Treatment with increasing amounts of serum resulted in a concentration-dependent increase in cell number. The stimulatory effect TGFα was greater with increasing concentrations of serum in the cell culture medium. When KGN cells were cultured in DMEM with 10% FBS, TGFα treatment almost doubled the KGN cell number (Figure 3A) compared to the FBS control. Similar to the stimulatory effect of TGFα on proliferation of KGN cells, treatment of COV434 cells with TGFα in serum-free DMEM resulted in a statistically significant increase in cell number compared to control (*P*<0.05) (Supplemental Figure 1).

AG1478, an EGFR kinase inhibitor; LY294002 a PI3K inhibitor; U0126, a MEK1 inhibitor; and rapamycin, a mTOR inhibitor, were used to determine the cellular signaling pathways involved in the TGFα-induced stimulation of KGN cell proliferation. AG1478 prevented the stimulatory effect of TGFα on cell proliferation, suggesting involvement of EGFR. LY294002 and rapamycin also significantly reduced TGFα-induced KGN cell proliferation, suggesting involvement of the PI3K/Akt pathway and mTOR pathways in this process. Surprisingly, U0126 treatment provoked a slight, but not statistically significant reduction in TGFα-induced KGN cell proliferation (Figure 3B).

**Figure 4:**
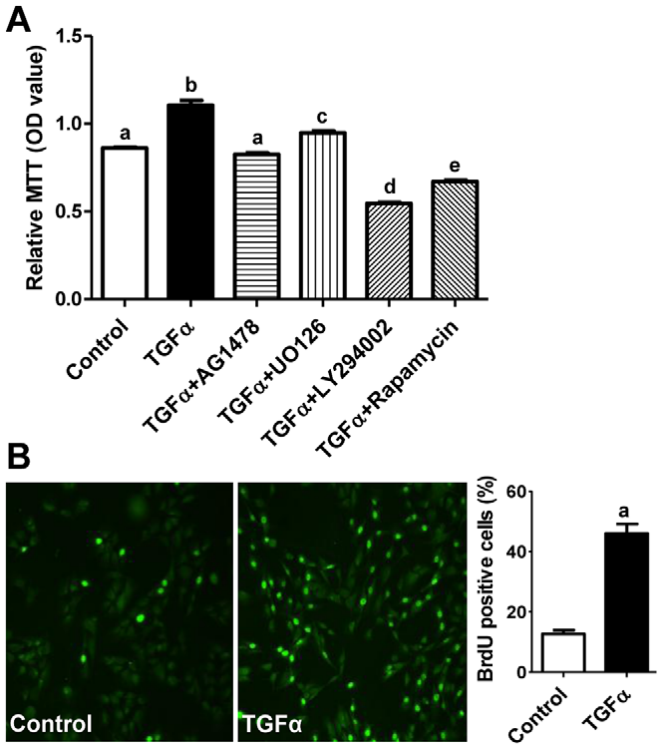
Effect of TGFα on KGN cell viability and DNA synthesis. **A**) KGN cells were treated with or without TGFα for 48 hours and cell viability was detected with MTT assay. Bars represent means ± SEM. Bars with different letters are significantly different (*P*<0.05) from each other. **B**) Effect of TGFα on KGN cell DNA synthesis. KGN cells were treated with or without TGFα for 36 hours and DNA synthesis was detected by BrdU incorporation. A representative image for BrdU immunofluorescence is shown for both control and TGFα treated groups. Magnification, 200×. Bars represent means ± SEM. “a”, significantly different (*P*<0.05) from the control group.

The MTT assay showed that, compared to control, treatment with TGFα significantly increased KGN cell viability (Figure 4A, *P*<0.05). Treatment with pathway-specific inhibitors reduced cell viability comparable to the extent that they reduced cell proliferation in TGFα treated cells (Figure 3B, Figure 4A). Moreover, treatment of KGN cells with TGFα significantly stimulated DNA synthesis in the KGN cells (*P*<0.05), as indicated by an increase in BrdU incorporation after TGFα treatment (Figure 4B).

**Figure 5:**
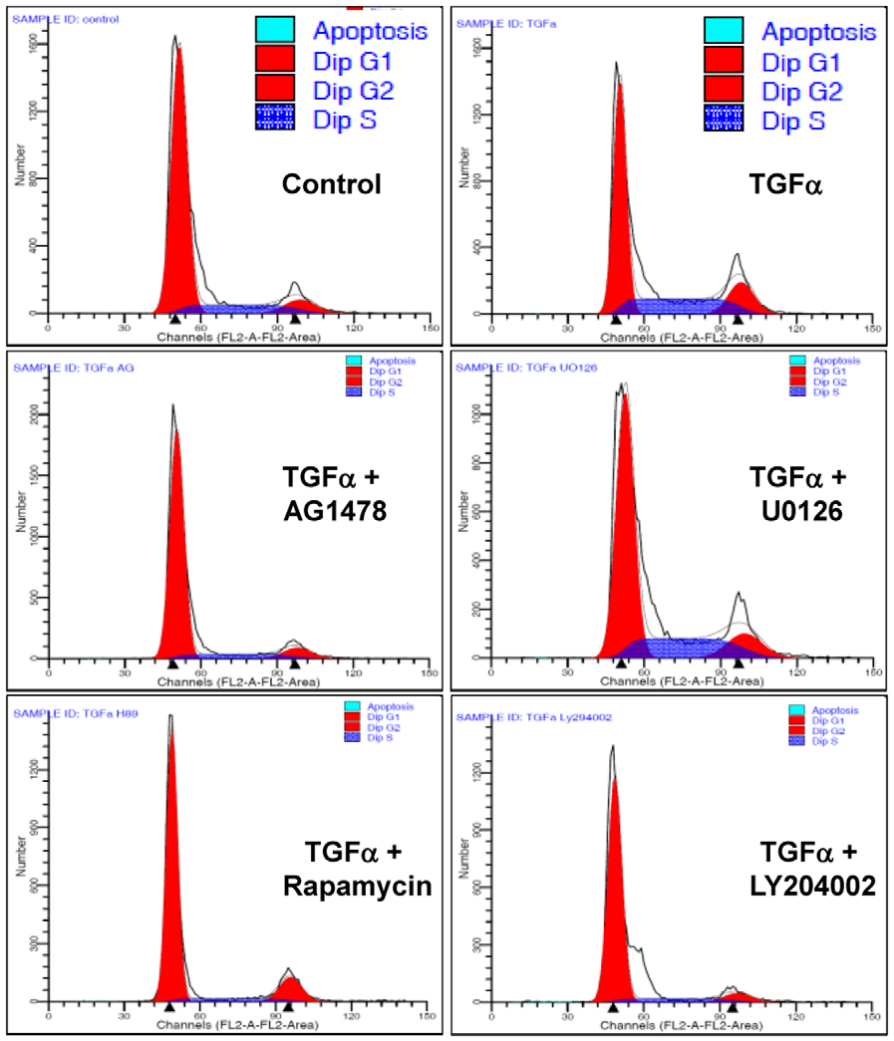
Effect of TGFα and kinase inhibitors on KGN cell cycle progression. KGN cells were treated without (Control) or with TGFα (10 ng/ml) and/or kinase inhibitors for 24 hours and cell cycle progression was determined by flow cytometry. AG1478 (100 nM): EGFR kinase inhibitor; U0126 (4 µM): MEK inhibitor; Rapamycin (20 nM): mTOR inhibitor; LY294002 (100 nM), PI3K inhibitor. Results are representative of three separate experiments.

Flow cytometry was used to evaluate the effect of TGFα on the GCT cell cycle progression and cell apoptosis. TGFα significantly increased the percentage of KGN cells in the S (*P*<0.001) and G2/M phases (*P*<0.05) and reduced the portion of cells in the G1 phase (*P*<0.001) (Figure 5 and Table 1). The effects of TGFα on the cell cycle was inhibited by treatment with AG1478, LY294002 or rapamycin, but not by treatment with U0126 (Figure 5), suggesting that the stimulatory effect of TGFα on KGN cell cycle progression was mediated by EGFR, PI3K/Akt and mTOR pathways. TGFα treatment did not affect apoptosis of KGN cells.

### TGFα stimulates KGN cell migration

**Figure 6:**
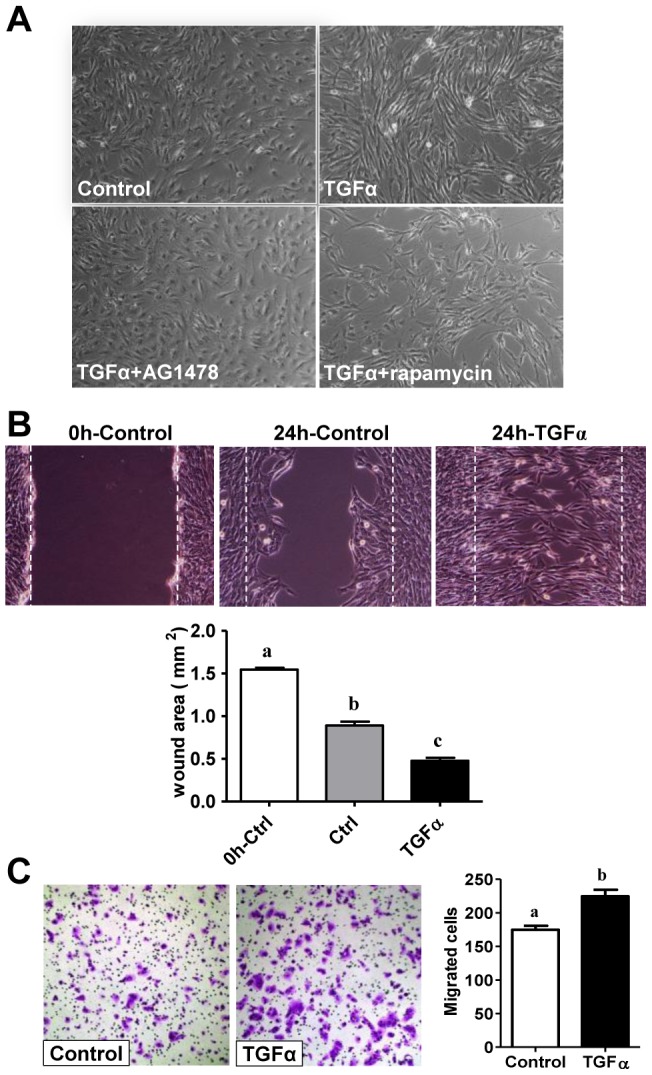
Effects of TGFα on KGN cell morphology and migration. **A**) Effect of TGFα on KGN cell morphology. TGFα treated cells become elongated and morphologically more fibroblast-like. Magnification, 200×. **B**) The would healing assay was performed to determine the effect of TGFα on KGN cell migration. The initial wound is highlighted by the white dashed line. The wound area was quantified with Microsuit™ FIVE software (Olympus American inc. Center Valley, PA). Bars represent mean areas ± SEM. Bars with different letters are significantly different (*P*<0.05) from each other. Magnification, 200×. **C**) Effects of TGFα on KGN cell migration analyzed with a Transwell migration assay system. Migrated cells were counted manually. Data are presented as means ± SEM. Bars with different letters are significantly (*P*<0.05) different from each other. Magnification, 100×.

We observed that treatment of KGN cells with TGFα not only increased cell number, but also changed cell morphology. Compared with control, treatment of KGN cells with 10 ng/ml of TGFα for 24 hours caused the cells to become spindle shaped. Cells treated with TGFα for 48 hours or longer were more organized into a sinuous pattern, indicating an increase in the mobility of KGN cells (Figure 6A). We then used a wound healing assay to determine whether TGFα was able to stimulate KGN cell migration. We found that TGFα treatment induced greater migration of KGN cells and significantly reduced the wound area (Figure 6B) compared with that of control (Figure 6B, *P*<0.001), indicating that TGFα is able to stimulate KGN cell migration. We used a transwell migration assay to corroborate this result. We found that TGFα treatment significantly increased the number of cells that migrated through the membrane of the transwell insert (*P*<0.05), confirming the capacity of TGFα to stimulate KGN cell migration (Figure 6C).

### TGFα regulates KGN cell actions via multiple pathways

**Figure 7:**
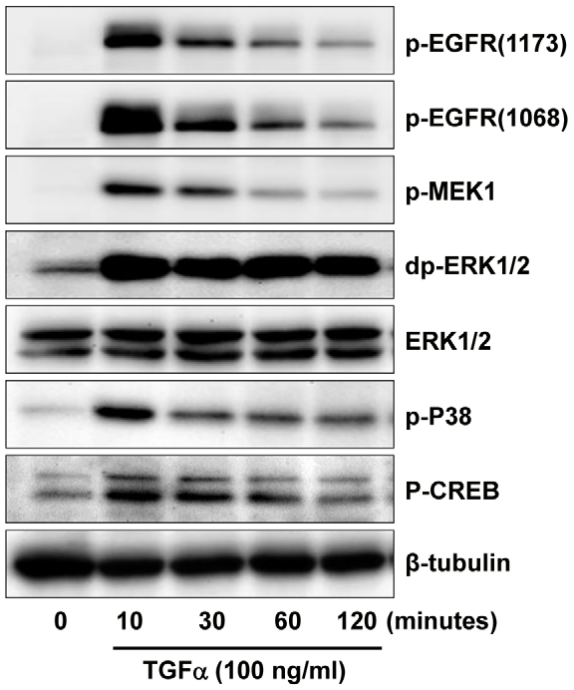
Effect of TGFα on the activation of MAPK signaling pathways in cultured KGN cells. KGN cells were incubated with or without 10 ng/ml of TGFα for 0, 10, 30, 60 or 120 minutes. Phosphorylation of proteins was detected with western blot. Results are representative of three separate experiments.

As mentioned above, the EGFR/PI3K/Akt and mTOR pathways may be involved in TGFα-induced KGN cell proliferation. We used AG1478, a TGFα receptor tyrosine kinase inhibitor, and pathway-specific kinase inhibitors to verify these observations. Treatment of KGN cells with TGFα induced phosphorylation of EGFR at tyrosine 1173 and tyrosine 1068 within 10 minutes of treatment (Figure 7). TGFα treatment also rapidly induced phosphorylation of MEK1 and ERK1/2, but did not affect the expression of total ERK1/2 (Figure 7). TGFα treatment also rapidly stimulated phosphorylation of P38 MAPK and CREB, suggesting that additional pathways are also activated in TGFα-stimulated KGN cells (Figure 7).

**Figure 8:**
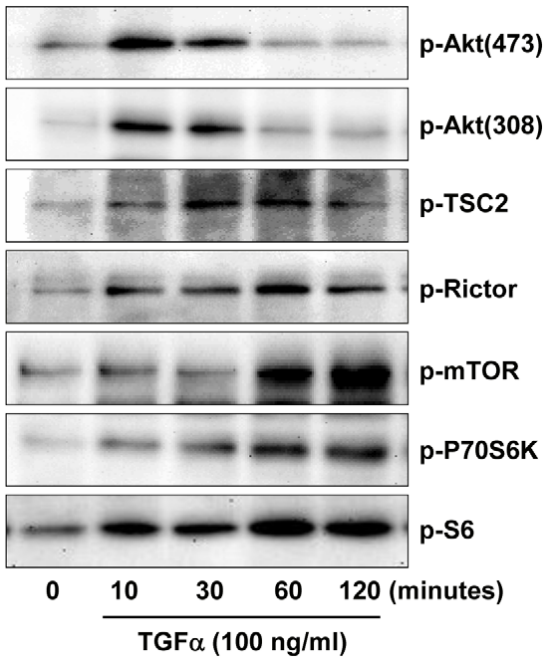
Effect of TGFα on the activation of PI3K/AKT and mTOR pathways in KGN cells. KGN cells were incubated with or without 10 ng/ml of TGFα for 0, 10, 30, 60 or 120 minutes. Phosphorylated and non-phosphorylated proteins were detected with western blot. Results are representative of three separate experiments.

We observed that TGFα rapidly stimulated the phosphorylation of Akt at both serine 473 and threonine 308 (Figure 8). Akt phosphorylation decreased at 30 minutes and returned to basal level 60 minutes after TGFα treatment (Figure 8). TGFα treatment also rapidly induced phosphorylation of mTORC1 pathway signaling components TSC2, rictor, mTOR, p70S6K, and its target ribosomal protein S6 (Figure 8). The activation of the mTORC1 pathway was sustained throughout the 120 minute incubation with TGFα. These results, in combination with observations in Figure 3 showing that inhibition of PI3K and mTOR inhibited TGFα-induced KGN cell proliferation, make it reasonable to suggest that PI3K/Akt and mTOR pathways are involved in TGFα-induced KGN cell proliferation.

**Figure 9:**
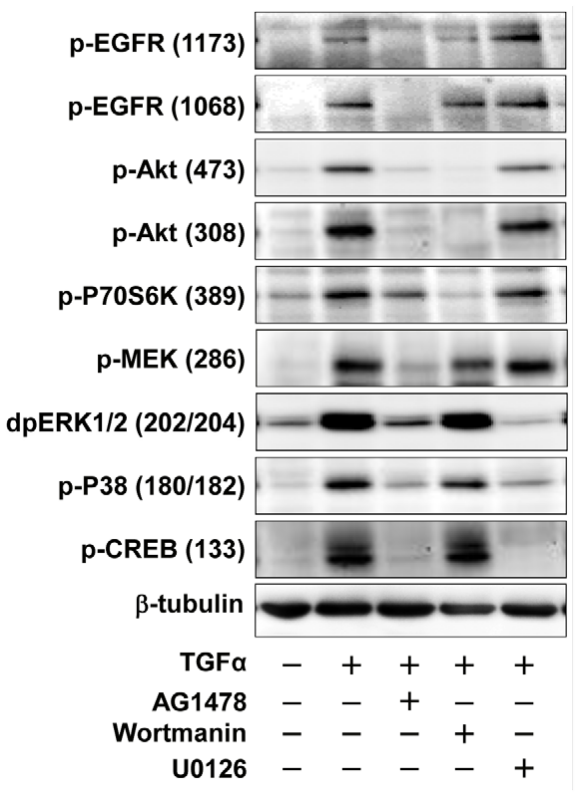
Effects of pathway specific kinase inhibitors on signaling pathways involved in the TGFα actions in KGN cells. KGN cells were pretreated with or without pathway inhibitors for 3 hours before treatment with TGFα for 10 minutes. Phosphorylated and non-phosphorylated proteins were detected by Western blot. Results are representative of three separate experiments.

Treatment with AG1478 totally prevented the effects of TGFα (30 minutes) on the activation of all the pathways examined, suggesting that EGFR is indispensable for mediating the actions of TGFα in KGN cells (Figure 9). Pretreatment of KGN cells with wortmannin blocked the effect of TGFα on the activation of Akt and p70S6K, but did not affect TGFα activation of EGFR, ERK1/2, P38 or CREB in the KGN cells (Figure 9). U0126 treatment did not affect TGFα-induced activation of EGFR, Akt, P70S6K, but it blocked ERK1/2 and CREB phosphorylation (Figure 9).

**Figure 10:**
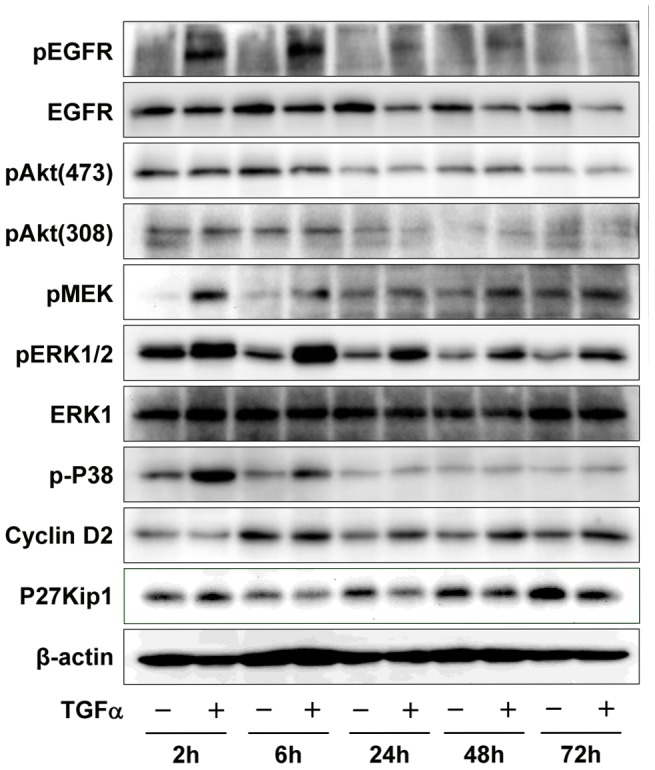
Long-term effects of TGFα on the activation of signaling pathways and expression of cell cycle regulators in KGN cells. KGN cells were incubated with or without TGFα (10 ng/ml) for 2, 6, 24, 48 or 72 hours. Phosphorylated and non-phosphorylated proteins were detected by Western blotting. Results are representative of three separate experiments.

Long-term treatment (2–72 hours) of KGN cells with TGFα led to sustained activation of EGFR, as evidenced by sustained tyrosine phosphorylation of EGFR. However, it also led to a reduction in the amount of EGFR in the KGN cells (Figure 10). This result is consistent with the idea that chronic stimulation causes the internalization and turnover of the EGFR [50]. TGFα-stimulated phosphorylation of Akt in KGN cells was transient and maintained for less than 60 minutes (Figure 8); whereas TGFα-stimulated phosphorylation of P38 and MEK was sustained for 6 hours (Figure 10). Surprisingly, TGFα-stimulated phosphorylation of ERK1/2 was sustained for more than 3 days (Figure 10). Treatment of KGN cells with TGFα for 24 hours significantly (*P*<0.01) increased cyclin D2 expression (relative intensities: 51±1.6 versus 67±1.6; for control and TGFα, respectively, mean ± SEM, n = 3) and suppressed p27Kip1 expression (relative intensities: 22±0.5 versus 15±0.7; for control and TGFα, respectively, mean ± SEM, n = 3) (Figure 10). The TGFα-induced increase in cyclin D2 and decrease in p27Kip1 in cultured KGN cells was sustained for at least 3 days (Figure 10).

## Discussion

Under physiologic conditions, the granulosa cell is regulated by reproductive hormones and growth factors in an endocrine, paracrine and autocrine manner [18], [19]. Any disruption in the regulatory pathways that function during normal ovarian development, folliculogenesis, and ovulation may result in uncontrolled granulosa cell proliferation and lead to malignant transformation. Several factors and cellular signaling pathways have been implicated in the initiation and progression of GCT. For example, studies with mutant mouse models show that a deficiency in α-inhibin expression contributes to initiation of GCT [51]. However, as patients with GCTs generally have high levels of circulating inhibins [2], [52], the role of inhibin in the development of GCTs in humans requires further evaluation. More recent studies with mouse models suggest that the loss of PTEN or activation of kRas and concomitant activation of Wnt/β-catenin signaling contributes to the initiation and progression of GCT [53], [54]. Despite the compelling GCT phenotypes, the relevance of these pathways in the initiation of juvenile or adult human GCTs has not been established. Recent somatic gain-of-function studies have shown that mutations in the transcription factor FOXL2 may be an important biomarker for the diagnosis of human ovarian GCTs [55]. Nevertheless, the mechanisms underlying the biologic activities and role of FOXL2 in the initiation, progression, recurrence and metastasis of ovarian GCT remain unknown. Our present study shows that the ErbB family of tyrosine kinase receptors is expressed in human granulosa tumor tissues and two human GCT cell lines. We observed that TGFα, via EGFR, promoted proliferation of KGN and COV434 cells and facilitated KGN tumor cell migration. Our data also indicate that multiple pathways mediate the stimulatory effects of TGFα on proliferation of the KGN GCT cells. Although the function of the ErbB family on ovarian follicle development, ovulation, and ovarian epithelial tumorigenesis have been extensively studied [20]–[21], [26]–[27], to our knowledge, this is the first direct evidence showing the function of EGF family of ligands, specifically TGFα, on GCT cell growth and possibly in GCT progression.

Mutation, overexpression of receptors, or constitutive activation of the downstream pathways of ErbB receptors are associated with many diseases, including ovarian cancer [56]. Expression of ErbB receptors on normal ovarian cells and on epithelial ovarian cancer cells has been well studied [26]–[27]. On the other hand, the results from two reports on the expression of ErbB family proteins in human GCTs are inconsistent. Furger et al. [48] showed that ErbB2 is expressed in the COV434 granulosa tumor cell line and in 6 of 12 human GCT tissue samples. Conversely, a more recent immunohistochemical study of 40 GCT cases reported that ErbB2 was undetectable in all 40 cases [49]. The reason for these differences is not clear since both studies used an ErbB2 antibody from the same source. Our study using another validated antibody showed that ErbB2 was present in KGN and COV434 cells and 5 of 5 patient samples. Furger et al. [48] also showed that 2 of 12 patient tumor samples expressed ErbB3, and 10 of 12 expressed ErbB4. However, Leibl et al. [49] reported that EGFR was present in 65% of the cases, and positive reactions for ErbB3 and ErbB4 were observed in 45% and 58% of the cases, respectively. Our present results in 5 human GCT cases and 2 GCT cell lines showed positive staining for ErbB3 and ErbB4. Based on these findings, it seems likely that GCTs will express multiple members of the ErbB family of receptors.

We observed that the expression patterns of ErbB receptors in the two GCT cell lines were different, with the COV434 cell line expressing higher levels of ErbB3 and ErbB4. Despite these differences TGFα stimulated proliferation of both cell lines. It is possible, however, that these two cell lines may respond differently to other members of the EGF family of ligands (e.g., heparin-binding EGF-like growth factor, amphiregulin, betacellulin, epiregulin, neuregulin) based on increased expression of ErbB3 and ErbB4 in COV434 cells. Evidence in other systems indicates the EGF family of ligands is capable of activating distinct ErbB receptors, signaling pathways, and biologic responses [57]–[58]. Additional experiments are warranted to test this possibility. Our data also showed that TGFα and EGF are expressed in both GCT cell lines. Expression of both receptors and ligands in GCT cells suggests that autocrine and/or paracrine mechanisms may be involved in TGFα regulation of GCT cell growth. It is well-known that activation of EGFR by TGFα or EGF leads to a rapid internalization of these receptors. The internalized EGFR is transported to early endosomes where receptor-ligand complexes are sorted for either degradation or recycling to the cell surface [50]. Results in the present study clearly show that the total EGFR level significantly decreased in KGN cells 6 hours after TGFα stimulation (Figure 10), suggesting that the internalization and recycling pathways are active in KGN cells.

Our current study demonstrates that TGFα stimulates the proliferation of KGN and COV434 cells. These findings are somewhat inconsistent with previously published results. Imai et al. [44] reported that exogenous EGF did not influence KGN cell proliferation. Unfortunately, it is not possible to directly compare the approaches in this study with the previous report due to a paucity of experimental detail in the previous report. One explanation for this discrepancy may be that TGFα is a more potent mitogen than EGF in KGN cells. It is also possible that in some circumstances, TGFα and EGF have different functions on certain cells [57], [59]. Moreover, we observed that stimulation of KGN cells with EGF did not promote KGN cell proliferation in serum-free medium; in contrast TGFα stimulated cell growth under serum-free conditions (Supplemental Figure 2). However, if the culture medium was supplemented with 10% of serum, both TGFα and EGF significantly stimulated KGN cell proliferation. Thus, other hormones or cytokines may also be required for EGF to optimally stimulate KGN cell proliferation. It seems that the presence of these unknown factor(s) may increase the sensitivity of KGN cells to TGFα and EGF stimulation.

GCTs are able to metastasize to many organs [9]–[17], but the mechanisms underlying GCT metastasis are largely unknown. It seems likely that hormones and growth factors may contribute to GCT metastasis. A recent report showed that KGN cells were capable of metastasis after implantation into nude mice [44]. The metastasis of the KGN cell tumor developed slowly and mimicked the characteristics of GCT metastasis reported previously [44], [60]. We found that TGFα had a profound effect on the morphology of KGN cells and significantly stimulated KGN cell migration. The morphological transition and enhanced migratory ability of TGFα-treated KGN cells suggests that TGFα may facilitate or promote GCT cell metastasis.

Although EGFR signaling pathways in ovarian epithelial cancer cells have been described [26]–[27], very little has been shown regarding the signaling pathways activated by TGFα and EGF in GCTs. Results in the present study suggest that TGFα rapidly activates the PI3K/Akt/mTOR and Raf/MEK/ERK1/2 signaling pathways. Our findings are partially consistent with Zhang et al., who recently studied the role of HOXA7 in the regulation of granulosa cell proliferation and showed that EGF transiently stimulated phosphorylation of ERK1/2 and Akt in KGN cells [61]. Our results showed that while TGFα stimulated a transient activation of Akt, it stimulated a sustained activation of ERK1/2; in fact TGFα-stimulated activation of ERK1/2 was sustained for more than 3 days (Figure 10). The duration of ERK activity has been implicated as a critical factor in cell fate decisions [62]–[63]. Whereas transient ERK activation causes cell proliferation, the overexpression of EGFR induced a sustained ERK activation leading to differentiation of PC12 neuronal cells [64]. In contrast, in CCL39 Chinese hamster lung fibroblasts it is sustained, but not transient, activation of ERK that is required for the proliferation of quiescent fibroblasts [65]. Immediate early genes downstream of ERK activation, such as Fos, Jun, Myc and Egr1, encode critical transcription factors for cell proliferation. Furthermore, sustained ERK activation causes phosphorylation and stabilization of the proteins encoded by these genes. For example, cyclin D1, which is important for S-phase entry and under the regulation of AP-1 (Jun and Fos proteins), is elevated and maintained by sustained ERK activation [63], [65]. The prolonged activation of ERK by TGFα in GCT cells suggests that TGFα might be a major regulator of GCT cell fate. However, treatment with U0126, which effectively inhibited ERK activation in response to TGFα, was unable to completely suppress the proliferation of KGN cells. The function of TGFα-induced sustained activation of ERK in GCT warrants further investigation.

Cyclin D2 and p27Kip1, which function in the G1-S phase transition, are critical cell cycle regulators in ovarian granulosa cells [66]. In the present study, treatment of KGN cells with TGFα hours significantly increased cyclin D2 expression and suppressed p27Kip1 expression. The changes in cyclin D2 and p27Kip1 may explain the increased number of KGN cells in the S and G2/M phases after TGFα treatment. The present in vitro findings with human GCT cells are supported by studies using mutant mouse models. Cyclin D2 deficient females mice are sterile because the granulosa cells are unable to proliferate normally in response to FSH [67]. Tumorigenesis in the α-inhibin deficient mice is accompanied by an increased expression of cyclin D2 and a decreased expression of p27Kip1 [68]. Mutant mice with double knockout of p27Kip1 and α-inhibin developed and succumbed to ovarian tumors more rapidly than α-inhibin knockout mice. However, cyclin D2 and α-inhibin double-knockout mice lived longer than mice lacking α-inhibin alone [69], suggesting that p27Kip1 acts cooperatively with inhibins to negatively regulate granulosa cell proliferation [68]–[69] and cyclin D2 may antagonize the tumor-suppressing actions of p27Kip1. Taken together, these findings suggest that TGFα may be involved in granulosa cell tumor initiation and progression. More experiments are required to reach a final conclusion.

The components of PI3K/AKT/mTOR signaling pathway are frequently overexpressed in human epithelial ovarian cancer and are attractive targets for epithelial ovarian cancer therapy. Phase I–II trials are currently evaluating mTOR inhibitors in ovarian cancer patients [70]–[71]. However, little is known about the expression and action of components of this pathway in GCT. One recent study examining 12 GCTs showed that mTOR was expressed in every tumor sample and phosphorylated mTOR was detected in 30% of the tumor tissues [72]. This study correlated the presence of mTOR with the expression of HIF1α and VEGF, both of which are important for tumor angiogenesis. In the present study, we demonstrated that TGFα, via EGFR, stimulated phosphorylation of mTOR and its downstream target proteins p70S6K and rpS6. This pathway appears to be critical for the actions of TGFα on KGN cell proliferation as our studies demonstrate that proliferation was disrupted by treatment with inhibitors of PI3K and mTOR. As expected, the activation of Akt and mTOR signaling in response to TGFα was rapid and the PI3K inhibitor blocked both AKT and mTOR signaling. However, Akt signaling was transient and returned to base line after 30 minutes while the phosphorylation of the mTOR targets p70S6K and rpS6 continued to increase over the 2 hour time course. This suggests that TGFα can maintain mTOR signaling independent of ongoing Akt signaling in KGN cells. In support of this idea, Fan et al. reported that in glioma cells the EGFR can activate mTOR independent of Akt by a mechanism involving protein kinase C [73]. While the cellular mechanism responsible for the sustained elevation in mTOR signaling in KGN cells awaits investigation, our study clearly demonstrates that the mTOR inhibitor rapamycin effectively suppressed TGFα-induced proliferation of KGN cells. Thus, mTOR-targeted therapy may represent a strategy for reducing vascularization of GCT [72] and inhibiting growth of GCTs that express TGFα and ErbB receptors. Furthermore, dual PI3K and mTOR inhibitors may provide additional therapeutic benefit [70], [71].

In summary, our present study suggests that TGFα, via ErbB receptors, promotes KGN GCT cell cycle progression, enhances tumor cell proliferation and facilitates GCT cell migration. Moreover, human GCT cells express the ligand TGFα and its ErbB receptors suggesting that components are available in human GCTs to promote the proposed actions of TGFα on GCT cell proliferation and facilitate tumor cell migration in an autocrine and/or paracrine manner. Our results also indicate that multiple signaling pathways are involved in TGFα regulation of GCT cell growth and migration.

## Supporting Information

Figure S1 **Effect of TGFα on the proliferation of COV434 granulosa tumor cells in vitro.**
(DOC)

Figure S2 **Effect of EGF on the proliferation of KGN cells in vitro.**
(DOC)

Table S1 **Oligonucleotide primer sequences used for RT-PCR.**
(DOC)
