# Supplementary material for: Transforming Growth Factor Alpha (TGFα) Regulates Granulosa Cell Tumor (GCT) Cell Proliferation and Migration through Activation of Multiple Pathways
Source: PLoS One. 2012 Nov 14;7(11):e48299. doi: 10.1371/journal.pone.0048299 (PMC3498304; doi:10.1371/journal.pone.0048299)
Supplement: Figure S1 — Effect of TGFα on the proliferation of COV434 granulosa tumor cells in vitro. (DOC) [file pone.0048299.s001.doc]

**Supplemental Figure S1. Effect of TGFα on the proliferation of COV434 granulosa tumor cells *in vitro*.** COV434 cells were plated DMEM containing 10% FBS. Upon reaching approximately thirty percent confluence, the media was changed to serum-free DMEM and COV434 cells were incubated in the presence or absence of TGFα (10 ng/ml) for 48 hours in a humidified 5% C0_2_/air cell culture incubator at 37°C. The cell number was determined with an Invitrogen Countess® Automated Cell Counter (Carlsbad, CA). Bars represent means ± SEM. *, p <0.05 vs 0 ng/ml TGFα.

*
