# Supplementary material for: Transforming Growth Factor Alpha (TGFα) Regulates Granulosa Cell Tumor (GCT) Cell Proliferation and Migration through Activation of Multiple Pathways
Source: PLoS One. 2012 Nov 14;7(11):e48299. doi: 10.1371/journal.pone.0048299 (PMC3498304; doi:10.1371/journal.pone.0048299)
Supplement: Figure S2 — Effect of EGF on the proliferation of KGN cells in vitro. (DOC) [file pone.0048299.s002.doc]

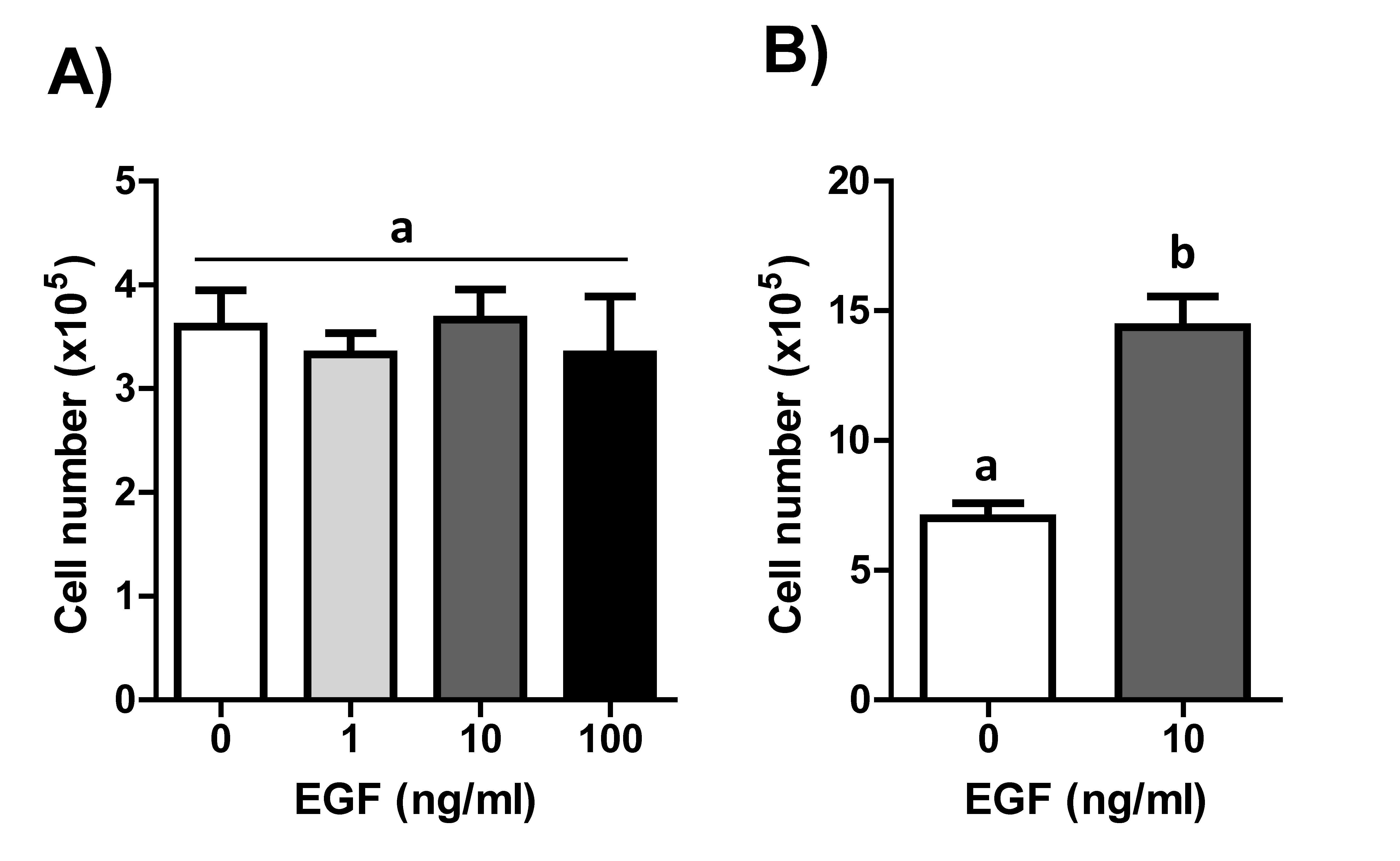


**Supplemental Figure S2. Effect of EGF on the proliferation of KGN cells *in vitro*.** KGN cells were plated DMEM containing 10% FBS. Upon reaching approximately thirty percent confluence, the media was changed to serum-free DMEM. **A)** KGN cells were incubated at 37°C in serum-free DMEM in the presence or absence of EGF (0-100 ng/ml) for 72 hours. **B**) KGN cells were treated for 72 hours in DMEM supplemented with 10% FBS in the presence or absence of 10 ng/ml of EGF. The cell number was determined with an Invitrogen Countess® Automated Cell Counter (Carlsbad, CA). Bars represent means ± SEM, n=3 experiments. Bars with different letters are significantly (p<0.05) different from each other. The results show that EGF is able to stimulate KGN cell proliferation only in the presence of serum.

Serum-free

10% Serum
