# Supplementary material for: Transforming Growth Factor Alpha (TGFα) Regulates Granulosa Cell Tumor (GCT) Cell Proliferation and Migration through Activation of Multiple Pathways
Source: PLoS One. 2012 Nov 14;7(11):e48299. doi: 10.1371/journal.pone.0048299 (PMC3498304; doi:10.1371/journal.pone.0048299)
Supplement: Table S1 — Oligonucleotide primer sequences used for RT-PCR. (DOC) [file pone.0048299.s003.doc]

**Supplemental Table S1 Oligonucleotide primer sequences used for RT-PCR.**
